# Oxolinic Acid (Pesticides/Veterinary Medicinal Products)

**DOI:** 10.14252/foodsafetyfscj.D-26-00014

**Published:** 2026-06-26

**Authors:** Food Safety Commission of Japan

## Abstract

Food Safety Commission of Japan (FSCJ) conducted a risk assessment of oxolinic acid (CAS No. 14698-29-4), an antimicrobial agent with a quinoline structure, based on results from submitted documents. For the fifth edition, additional residue data for crops (including taro and broccoli) were submitted by the Consumer Affairs Agency. The data used in the assessment include fate in animals (including rats) and humans, fate in plants (including paddy rice and napa cabbage), residues in crops, residues in livestock products (including cattle and pigs), subacute toxicity (rats, mice, and dogs), chronic toxicity (dogs), combined chronic toxicity/carcinogenicity (rats), carcinogenicity (mice), two-generation reproductive toxicity (rats), developmental toxicity (rats and rabbits), and genotoxicity. Major adverse effects of oxolinic acid were observed in the body weight (suppressed weight gain), the testes (interstitial cell hyperplasia in rats), the ovaries (organ weight increase in rats), excitatory neurological symptoms, and behavioral changes (**Table 1**). No adverse effects were observed on either fertility, teratogenicity, or biologically significant genotoxicity. The lowest NOAEL for potential adverse effects after a single oral administration of oxolinic acid was 6 mg/kg bw from the results of the acute neurotoxicity study in rats (**Table 2**). FSCJ established an acute reference dose (ARfD) of 0.06 mg/kg bw by applying a safety factor of 100 to this NOAEL.

## Conclusion in Brief

Food Safety Commission of Japan (FSCJ) conducted a risk assessment of oxolinic acid (CAS No. 14698-29-4), an antimicrobial agent with a quinoline structure, based on results from submitted documents. For the fifth edition, additional residue data for crops (including taro and broccoli) were submitted by the Consumer Affairs Agency.

The data used in the assessment include fate in animals (including rats) and humans, fate in plants (including paddy rice and napa cabbage), residues in crops, residues in livestock products (including cattle and pigs), subacute toxicity (rats, mice, and dogs), chronic toxicity (dogs), combined chronic toxicity/carcinogenicity (rats), carcinogenicity (mice), two-generation reproductive toxicity (rats), developmental toxicity (rats and rabbits), and genotoxicity.

Major adverse effects of oxolinic acid were observed in the body weight (suppressed weight gain), the testes (interstitial cell hyperplasia in rats), the ovaries (organ weight increase in rats), excitatory neurological symptoms, and behavioral changes (Table 1). No adverse effects were observed on either fertility, teratogenicity, or biologically significant genotoxicity.

**Table 1.** *Levels relevant to toxicological evaluation of oxolinic acid*

Species	Study	Dose (mg/kg bw per day)	NOAEL (mg/kg bw per day)	LOAEL (mg/kg bw per day)	Critical endpoints
Rat	30-day subacute toxicity study	0, 125, 250, 500, 1 000	M: 125 F: -	M: 250 F: 125	M/F: Increased absolute and /or relative weights of the adrenal gland
	90-day subacute toxicity study	0, 100, 300, 1 000, 3 000 ppm	M: 17.2 F: 6.48	M: 62.2 F: 19.9	M: Suppressed body weight gain, decrease in TP, decrease in Glob, etc. F: Decrease in Glu, etc.
		M: 0, 5.68, 17.2, 62.2, 204 F: 0, 6.48, 19.9, 77.4, 264			
	Six-month subacute toxicity study	0, 1 000, 3 000, 10 000, 30 000 ppm	M: - F: 0.08 (g/kg bw/day)	M: 0.06 F: 0.31 (g/kg bw/day)	M: Decrease in WBC, etc. F: Suppressed body weight gain, etc.
		M: 0, 0.06, 0.23, 0.79, 2.72 F: 0, 0.08, 0.31, 1.25, 3.60 (g/kg bw/day)			
	90-day subacute neurotoxicity study	0, 50, 300, 1 800 ppm	M: 19.4 F: 3.87	M: 132 F: 24.4	F/M: Excitatory neurological symptoms and behavioral changes, etc.
		M: 3.24, 19.4, 132 F: 3.87, 24.4, 175			
	Two-year combined chronic toxicity/carcinogenicity study	0, 30, 100, 300, 1 000 ppm	M: 3.60 F: 13.2	M: 10.9 F: 49.1	M: Red ocular discharge, increase in food intake F: Suppressed body weight gain, increase in food intake, emaciation, etc. (Increased incidence of interstitial cell tumors of the testes)
		M: 0, 1.06, 3.60, 10.9, 37.6 F: 0, 1.28, 4.38, 13.2, 49.1			
	Two-generation reproductive toxicity study	0, 50, 150, 500 ppm	Parents PM: 3.41 PF: 12.1 F_1_M: - F_1_F: 13.8 Offspring F_1_M: 10.3 F_1_F: 12.1 F_2_M: 41.2 F_2_F: 46.9	Parents PM: 10.3 PF: 41.8 F_1_M: 4.11 F_1_F: 46.9 Offspring F_1_M: 43.7 F_1_F: 41.8 F_2_M: - F_2_F: -	Parents: Suppressed body weight gain, etc. Offspring: Suppressed body weight gain (No effect on fertility was observed)
		PM: 3.41, 10.3, 34.7 PF: 3.91, 12.1, 41.8 F1M: 4.11, 12.4, 41.2 F1F: 4.49, 13.8, 46.9			
	Two-generation reproductive toxicity study ∙ additional study	0, 15, 30 ppm	Parents and offspring PM: 2.18 PF: 2.44 F_1_M: 2.52 F_1_F: 2.82	Parents and offspring PM: - PF: - F_1_M: - F_1_F: -	Parents: No toxicity Offspring: No toxicity (No effect on fertility was observed)
		PM: 1.07, 2.18 PF: 1.19, 2.44 F1M: 1.25, 2.52 F1F: 1.41, 2.82			
	Developmental toxicity study (the 1^st^ study)	0, 3, 30, 150	Dams: 3 Fetuses: 150	Dams: 30 Fetuses: -	Dams: Suppressed body weight gain Fetuses: No toxicity (No teratogenicity was observed)
	Developmental toxicity study (the 2^nd^ study)	0, 125, 250, 500, 1 000	Dams: 250 Offspring: 500	Dams: 500 Offspring: 1 000	Dams: Cannibalism of offspring, decrease in nursing rate Offspring: Low body weight (No teratogenicity was observed)
Mouse	90-day subacute toxicity study	0, 100, 300, 1 000, 3 000 ppm	M: 34.7 F: 47.1	M: 145 F: 184	F/M: Suppressed body weight gain, increase in food intake, decrease in food efficiency, emaciation/smaller body size, etc.
		M: 0, 11.2, 34.7, 145, 507 F: 0, 13.8, 47.1, 184, 493			
	18-month carcinogenicity study	0, 50, 150, 500 ppm	M: 15.2 F: 5.33	M: 59.7 F: 15.7	M: Skin lesions, increased mortality rate, suppressed body weight gain, etc. F: Suppressed body weight gain, decrease in ood efficiency (No carcinogenicity observed)
		M: 0, 4.86, 15.2, 59.7 F: 0, 5.33, 15.7, 57.9			
Rabbit	Developmental toxicity study	0, 250, 500, 1 000, 2 000	Dams: 2 000 Fetuses: 2 000	Dams: - Fetuses: -	Dams: No toxicity Fetuses: No toxicity (No teratogenicity observed)
Dog	90-day subacute toxicity study	M: 0, 8, 40, 200 F: 0, 8, 40, 200	M: 8 F: 8	M: 40 F: 40	M: Suppressed body weight gain, decrease in Glob F: Suppressed body weight gain
	One-year chronic toxicity study	M: 0, 8, 40, 200 F: 0, 8, 40, 200	M: 8 F: 8	M: 40 F: 40	M: Corneal white spots F: Corneal white spots, suppressed body weight gain
ADI			NOAEL: 2.18 SF: 100 ADI: 0.021		
The critical study for setting ADI			Two-generation reproductive toxicity study (rat)		

ADI, Acceptable daily intake; Glob, Globulin; Glu, Glucose; NOAEL, No-observed-adverse-effect level; SF, Safety factor; WBC, White blood cell count

-: NOAEL or LOAEL could not be specified.

The adverse effect observed at LOAEL

The mode of action was considered to be non-genotoxic, and it was deemed possible to establish a threshold for evaluation, although increased incidences of interstitial cell tumor were observed in rat testes.

Based on these results, oxolinic acid (parent compound only) was identified as the substance relevant for the residue definition in dietary risk assessment for agricultural products.

The lowest no-observed-adverse-effect level (NOAEL) was 2.18 mg/kg bw per day from a two-generation reproductive toxicity study in rats among the studies examined. FSCJ established an acceptable daily intake (ADI) of 0.021 mg/kg bw per day by applying a safety factor of 100 to the NOAEL.

The microbiological ADI of 0.071 mg/kg bw per day was calculated using the formula specified in the VICH guidelines.

Since the microbiological ADI was higher than the toxicological ADI, the latter value of 0.021 mg/kg bw per day was considered appropriate for setting the residue limit for oxolinic acid.

The lowest NOAEL for potential adverse effects after a single oral administration of oxolinic acid was 6 mg/kg bw from the results of the acute neurotoxicity study in rats (Table 2). FSCJ established an acute reference dose (ARfD) of 0.06 mg/kg bw by applying a safety factor of 100 to this NOAEL.

**Table 2.** *Potential adverse effects of a single oral administration of oxolinic acid*

Species	Study	Dose (mg/kg bw or mg/kg bw/day)	Endpoints relevant to setting NOAEL and ARfD^1^^)^ (mg/kg bw or mg/kg bw/day)
Rat	Acute toxicity study	F/M: 0, 20, 50, 200, 500, 630, 780, 1 000	F/M: 20 F/M: Increased spontaneous locomotor activity
	Acute neurotoxic study	F/M: 0, 6, 30, 150	F/M: 6 F/M: Increased spontaneous locomotor activity
	Six-month subacute toxicity study	M: 0, 0.06, 0.23, 0.79, 2.72 F: 0, 0.08, 0.31, 1.25, 3.60 (g/kg bw/day)	M: 0.06 F: 0.08 (g/kg bw/day) F/M: Neurological symptoms, etc.
	Developmental toxicity study (the 1^st^ study)	0, 3, 30, 150	Dams: 30 Dams: Self-biting behavior, suppressed body weight gain, decrease in food intake, etc.
Mouse	Acute toxicity study	F/M: 0, 10, 30, 800, 1 200, 1 800, 2 700, 4 000, 6 000	F/M: 10 F/M: Increased spontaneous locomotor activities, kyphotic posture
Dog	90-day subacute toxicity study	F/M: 0, 8, 40, 200	F/M: 40 F/M: Corneal white spots
	One-year chronic toxicity study	F/M: 0, 8, 40, 200	F/M: 8 F/M: Corneal white spots
ARfD			NOAEL: 6 SF: 100 ARfD: 0.06
The critical study for setting ARfD			Acute neurotoxicity study (rat)

ARfD, Acute reference dose; NOAEL; No-observed-adverse effect level; SF; Safety factor

^1)^ The adverse effect observed at LOAEL
